# Multi-layer cell-free scaffolds for osteochondral defects of the knee: a systematic review and meta-analysis of clinical evidence

**DOI:** 10.1186/s40634-021-00377-4

**Published:** 2021-07-30

**Authors:** Angelo Boffa, Luca Solaro, Alberto Poggi, Luca Andriolo, Davide Reale, Alessandro Di Martino

**Affiliations:** Clinica Ortopedica E Traumatologica 2, IRCCS Istituto Ortopedico Rizzoli, Via Giulio Cesare Pupilli, 1 - 40136 Bologna, Italy

**Keywords:** Cartilage, Multi-layer, Scaffold, Osteochondral, Cell-free, Knee

## Abstract

**Purpose:**

The aim of this study was to analyze the clinical results provided by multi-layer cell-free scaffolds for the treatment of knee osteochondral defects.

**Methods:**

A systematic review was performed on PubMed, Web of Science, and Cochrane to identify studies evaluating the clinical efficacy of cell-free osteochondral scaffolds for knee lesions. A meta-analysis was performed on articles reporting results of the International Knee Documentation Committee (IKDC) and Tegner scores. The scores were analyzed as improvement from baseline to 1, 2, and ≥ 3 years of follow-up. The modified Coleman Methodology Score was used to assess the study methodology.

**Results:**

A total of 34 studies (1022 patients) with a mean follow-up of 35 months was included. Only three osteochondral scaffolds have been investigated in clinical trials: while TruFit® has been withdrawn from the market for the questionable results, the analysis of MaioRegen and Agili-C™ provided clinical improvements at 1, 2, and ≥ 3 years of follow-up (all significantly higher than the baseline, *p* < 0.05), although with a limited recovery of the sport-activity level. A low rate of adverse events and an overall failure rate of 7.0% were observed, but the overall evidence level of the available studies is limited.

**Conclusions:**

Multi-layer scaffolds may provide clinical benefits for the treatment of knee osteochondral lesions at short- and mid-term follow-up and with a low number of failures, although the sport-activity level obtained seems to be limited. Further research with high-level studies is needed to confirm the role of multi-layer scaffold for the treatment of knee osteochondral lesions.

## Background

Knee osteochondral lesions have always represented a problem for the orthopedic surgeon because of the poor regenerative potential of the cartilage tissue coupled with the challenge of concomitantly addressing the affected subchondral bone [32, 61]. Several techniques have been developed over the years to address knee osteochondral lesions, in order to relieve pain, restore function and possibly delay osteoarthritis (OA) onset [14]. Traditional surgical approaches consist of autologous or allogenic osteochondral tissue transplantation to provide an immediate viable tissue at the lesion site [23, 25, 31]. These techniques demonstrated promising results up to long-term follow-up, but they also showed several drawbacks, such as a significant donor site morbidity for autologous osteochondral transplantation (OAT), and high cost, limited availability, and contamination risk for osteochondral allograft transplantation (OCA) [21, 31, 75]. Also, chondrocyte-based regenerative treatments developed to overcome these limitations did not provide an optimal solution for osteochondral lesions [26, 42, 54]. The reasons might be found in the complexity of cartilage-bone interface and the differences between cartilage and subchondral bone layers, including both biological and biomechanical properties. In this light, an optimal treatment should aim at addressing the entire osteochondral unit [53, 61].

Progress in the field of biomaterials has led to the development of various scaffolds to address the entire osteochondral unit, based on the rationale of reproducing the different biological and functional requirements for the growth of both bone and cartilage tissues [1, 35, 57]. In this regard, multi-layer cell-free osteochondral scaffolds have been introduced with the aim to provide a biomimetic and biodegradable three-dimensional structure that favors subchondral bone and cartilage-like tissue regeneration [22, 50, 51]. They showed a potential to act as stimuli for the differentiation of resident bone marrow stromal cells, by inducing an “in situ” tissue regeneration that allows a durable osteochondral tissue without the need for any cell augmentation [45, 52]. From a clinical point of view, these scaffolds showed to be easy to handle and exploitable in one-step procedures, avoiding issues related to cell manipulation and culture [44]. However, among the different solutions explored in the preclinical setting, only few cell-free multi-layer scaffolds have currently been investigated in clinical trials, and their results and effectiveness are still debated.

The aim of this study was to review the available literature and to analyze the clinical results provided by multi-layer cell free scaffolds for the treatment of knee osteochondral defects.

## Materials and methods

**Fig. 1:**
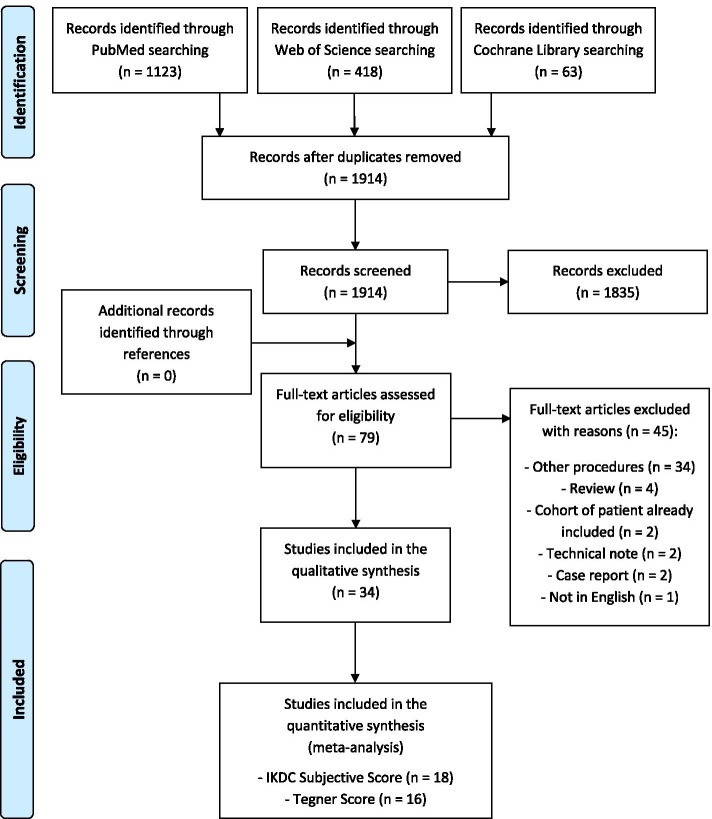
PRISMA (Preferred Reporting Items for Systematic Reviews and Meta-Analyses) flowchart of the study selection process

A systematic review and meta-analysis were performed on the literature of cell-free osteochondral scaffold implantation for knee lesions. The search was conducted on three electronic databases (PubMed, Web of Science, and Cochrane) on May 10, 2021, with no time limitation and without any filter, using the following string: (osteochondral) AND (scaffold OR matrix OR implant) AND (knee). The PRISMA (Preferred Reporting Items for Systematic Reviews and Meta-Analysis) guidelines were used [59]. A flowchart of the studies selection for qualitative and quantitative data synthesis is reported in Fig. 1. The screening process and analysis were conducted separately by 2 independent observers (LS and AP). In the first step, the articles were screened by title and abstract and the following inclusion criteria for relevant articles were used: clinical studies, written in the English language, on cell-free osteochondral scaffolds for the treatment of osteochondral lesions of the knee. Exclusion criteria were articles written in other languages, pre-clinical studies, studies reporting other osteochondral procedures such as cell-based scaffolds, OAT, OCA, case reports, and reviews. In the second step, the full texts of the selected articles were retrieved and screened, with further exclusions according to the previously described criteria. Reference lists from the selected papers and from the reviews of the field, found with the first and second screening, were also checked, and all selected studies were included in the qualitative data synthesis.

For the included studies, relevant data (year of publication, study design, number of patients evaluated, patient sex, age, and BMI, lesion size, lesion location, lesion grade, type of scaffold, scores reported, final follow-up, overall results) were extracted from article texts, tables, and figures, and then collected in a database with consensus of the two observers, to be analyzed for the purposes of the present study. To assess the methodological quality of the collected data, the Coleman Methodology Score (CMS), modified by Kon et al. [48] to better suit the cartilage repair field, was determined for each study. Two reviewers (LS and AP) independently evaluated the studies, and discrepancies were resolved through discussion and consensus with a third author (AB). Safety was evaluated through the reported adverse effects, while the failure rate was calculated through the documented surgical failures (patients requiring a joint replacement or a new surgery related to the scaffold implantation). Finally, the articles reporting clinical outcomes were selected and included in the meta-analysis. The scores were analyzed as improvement from baseline to 1, 2, and ≥ 3 years of follow-up, in order to investigate clinical results over time of cell-free osteochondral scaffold implantation. The articles included in the systematic review were excluded from the meta-analysis in the following cases: the same survey was reported at different follow-up times and the most recent articles also reported the intermediate follow-up results; mean basal scores or follow-up scores (including standard deviation) not reported; articles on multi-layer scaffolds withdrawn from the market.

### Statistical analysis

The statistical analysis and the Forest plot were carried out according to Neyeloff et al. [60] using Microsoft Excel. The comparisons among the follow-up times was based on the analysis of variance of the difference between basal and follow-up score (MD) [58]. With no heterogeneity, the estimation of the MD and its 95% confidence interval was based on fixed effect analysis of variance; the random effect model was preferred otherwise. *P*-value of 0.05 was used as the level of statistical significance. Statistical heterogeneity was evaluated by t using Cochran's Q statistic and I^2^ metric and was considered significant when I^2^ > 25%.

## Results

**Fig. 2:**
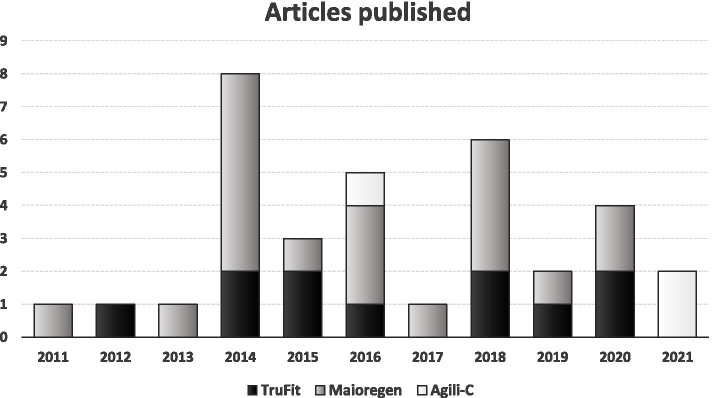
Number of articles per year dealing with cell-free osteochondral scaffolds and reporting clinical results for the treatment of knee lesions

The search identified 1914 records after duplicates removal, whose titles and abstracts were screened and selected according to the inclusion/exclusion criteria: 1835 records were excluded and a total of 79 full-text articles were assessed for eligibility; 45 full-text articles were further excluded (Fig. 1). Thus, a total of 34 studies were included in the qualitative data synthesis and reported in detail in Table 1. Since the first reports in 2011, beside a peak in 2014 (8 articles), the publication trend was stable over time (Fig. 2).

**Table 1:** Characteristics of the included studies

Author Journal Year	Study Design	Evaluated Patients	Age (years) Sex (M/F) BMI (Kg/m^2^)	Mean F-UP (months)	Treated Disease	Size Location Classification	Scaffold	Results and Failures	CMS
Kon et al AJSM 2021 [39]	Prospective Case Series	86	37.4 ± 10.0 60 M/26F 26.1 ± 3.5	24	Chondral and osteochondral lesions in mild to moderate OA	3.0 ± 1.7 cm^2^ 44 MFC/15 LFC/ 13 Trochlea/14 multiple ICRS III—IV	Agili-C™ (CartiHeal)	Significant improvement of all clinical scores. MRI showed a significant increase in defect filling over time. Eight failures	54
Van Genechten et al Cartilage 2021 [72]	Prospective Case Series	13	33.5 ± 8.9 10 M/3F 25.3 ± 3.4	36	Chondral and osteochondral lesions in mild to moderate OA	2.6 ± 1.7 cm^2^ 6 MFC/ 2 LFC/ 5 Trochlea ICRS III—IV	Agili-C™ (CartiHeal)	Statistically significant improvement of all clinical scores evaluated at 36 months. No failures	47
Guerin et al Orthop Traumatol Surg Res 2020 [34]	Retrospective Case Series	17	28 ± 9 9 M/8 F -	46	Osteochondral lesions	4.5 ± 1.4 cm^2^ 12 MFC/4 LFC/1 Tibia -	MaioRegen (Finceramica)	Good clinical outcomes in large knee osteochondral defects without correlation with MRI results. Failures not reported	34
Sessa et al Cartilage 2020 [68]	Prospective Case Series	20	16.2 ± 1.4 14 M/6F 21 ± 2.1	72	Osteochondral lesions in juvenile OCD	3.2 ± 1.8 cm^2^ 9 MFC/8 LFC/3 Trochlea ICRS III—IV	MaioRegen (Finceramica)	Clinical improvement stable over time with a high survival rate, although with persisting abnormal MRI findings. No failures	51
Shivji et al J. Orthop 2020 [69]	Prospective Case Series	11	46 ± 13.7 4 M/7F -	121	Chondral and osteochondral lesions	4.3 cm^2^ (2.9—8.0) 35 plugs implanted: 17 MFC/4 LFC/1 Patella 13 Trochlea ICRS III-IV	TruFit® (Smith&Nephew)	No statistically significant improvements in any clinical score evaluated also confirmed by MRI results. Four failures	45
Wang et al Cartilage 2020 [74]	Retrospective Comparative Study	66	42.9 ± 12.8 37 M/29F 26.7 ± 7.7	40	Chondral and osteochondral lesions	3 ± 1.7 cm^2^ 36 MFC/15 LFC/ 15 Trochlea ICRS III-IV	TruFit® (Smith&Nephew) vs Microfracture	Activity level and MRI appearance in the scaffold group were superior than microfracture group. One failure	63
D'Ambrosi et al Int Orthop 2019 [13]	Prospective Case Series	21	51.3 ± 10.7 14 M/7F 27.2 ± 3.3	60	Chondral and osteochondral lesions	- - ICRS IV	TruFit® (Smith&Nephew)	Good clinical outcome with stable results at long-term F-UP. No correlations with MRI results. Two failures	49
Sessa et al J Clin Med 2019 [67]	Prospective Case Series	22	39.0 ± 8.2 19 M/3F 25.0 ± 2.2	60	Chondral and osteochondral lesions in early OA	3.2 ± 1.9 cm^2^ 10 MFC/9 LFC/ 1 Patella/6 Trochlea/ 5 multiple ICRS III—IV	MaioRegen (Finceramica)	Significant clinical improvement and stable outcome with low complication and failure rates. Two failures	44
Azam et al J. Orthop 2018 [4]	Retrospective Case Series	10	35.2 (22–49) 7 M/3F -	24	Chondral and osteochondral lesions	- 10 MFC/2 LFC -	TruFit® (Smith&Nephew)	All patients showed a significant but small clinical improvement. Failures not reported	24
Bugelli et al Musculoskelet Surg 2018 [9]	Retrospective Case Series	5	64.4 (38–80) 4 M/1F -	71	Ostechondral lesions	- 7 MFC/ 1Trochlea ICRS III-IV	TruFit® (Smith&Nephew)	Clinical and radiological results significantly improve in a longer F-UP time No failures	33
Condello et al BioMed Research International 2018 [12]	Prospective Case Series	26	43.8 ± 11.2 18 M/8F 27.3 ± 4.3	35	Osteochondral lesions in early OA	- 17 MFC/3 Patella/ 6 Trochlea ICRS III – IV	MaioRegen (Finceramica)	Clinical outcomes showed significant improvement in 69% of the patients, although no correlations with radiological images results were found. No failures	46
Kon et al KSSTA 2018 [40]	Randomized Controlled Trial	51	34.0 ± 10.9 36 M/15F 25.6 ± 3.3	24	Chondral and osteochondral lesions	3.4 ± 1.5 cm^2^ 37 FC/12 Patella/ 2 Trochlea ICRS III—IV	MaioRegen (Finceramica) vs Microfracture	A statistically significant improvement of all clinical scores was described from basal evaluation to 2 years F-UP. Two failures	67
Mathis et al KSSTA 2018 [56]	Prospective Case Series	14	33.1 ± 10.7 11 M/3F 27 ± 4	12	Osteochondral lesions	1.0–3.5 cm^2^ 8 MFC/2 LFC/ 2 Patella/2 Trochlea ICRS III—IV	MaioRegen (Finceramica)	Significant clinical improvement at 1-year F-UP although an ongoing defect filling was found at the same F-UP. Failures not reported	27
Perdisa et al AJSM 2018 [64]	Prospective Case Series	27	25.5 ± 7.7 19 M/8F 23.0 ± 2.7	60	OCD lesions	3.4 ± 2.2 cm^2^ 17 MFC/10 LFC ICRS III—IV	MaioRegen (Finceramica)	Significant improvement of clinical scores at each F-UP, although not correlated with MRI findings. No failures	46
Perdisa et al AJSM 2017 [62]	Prospective Case Series	34	30 ± 10 18 M/16F 24 ± 3	24	Chondral and osteochondral lesions	2 ± 1 cm^2^ 34 Patella ICRS III—IV	MaioRegen (Finceramica)	A statistically significant improvement in all scores was observed at 12–24 months of F-UP. No failures	48
Berruto et al Knee 2016 [7]	Prospective Case Series	11	52.1 ± 9.6 5 M/6F 23.9 ± 1.6	24	Late-stage osteonecrosis of the knee	3.47 ± 1.75 cm^2^ 11 MFC 11 SPONK	MaioRegen (Finceramica)	Clinical scores improved significantly at 1 year of F-UP, remaining stable at 2 years. Two failures	33
Brix et al Int Orthop 2016 [8]	Prospective Case Series	8	37 (15–51) 6 M/2F -	24	Osteochondral lesions	2.07 cm^2^ (1.5–3.75) 5 MFC/3 LFC ICRS III—IV	MaioRegen (Finceramica)	Clinical scores improved without reaching statistical significance at 2 years of F-UP. T2 mapping described a limited quality of repair cartilage tissue. No failures	45
Christensen et al KSSTA 2016 [11]	Prospective Case Series	6	- - -	30	Osteochondral lesions	- 3 MFC/1 Patella -	MaioRegen Finceramica)	A significant clinical improvement was described, while MRI showed no improvement at any time point. Two failures	48
Dell’Osso et al Musculoskelet Surg 2016 [17]	Retrospective Case Series	31	60.6 (32–79) 16 M/15F -	48	Osteochondral lesions	- 43 TruFit plugs: 39 MFC/3 LFC/1 Trochlea -	TruFit® (Smith&Nephew)	Good clinical outcome at final F-UP. MRI showed partial integration of the scaffolds always with centripetal mode. One failure	27
Kon et al Injury 2016 [47]	Prospective Comparative Study	21	31.0 ± 8.6 17 M/4F 26.2 ± 3.4	12	Chondral and osteochondral lesions	2.5 ± 1.7 cm^2^ 11 MFC/4 LFC/ 6 Trochlea ICRS III—IV	Agili-C™ (CartiHeal) Tapered	A statistically significant improvement in all clinical scores evaluated without significant differences between the two groups No failures in the tapered group, while 8 failures in the Cylindrical group	46
		76	31.7 ± 7.8 62 M/14F 25.1 ± 3.4	12	Chondral and osteochondral lesions	1.7 ± 1.0 cm^2^ 45 MFC/25 LFC/ 6 Trochlea ICRS III—IV	Agili-C™ (CartiHeal) Cylindrical		
Dhollander et al Acta Orthop. Belg 2015 [18]	Prospective Case Series	20	31.7 (17–53) 8 M/12F -	34	Chondral and osteochondral lesions	0.83 cm^2^ (0.38–1.58) 8 MFC/4 LFC/ 5 Patella/3 Trochlea ICRS III—IV	TruFit® (Smith&Nephew)	Significant clinical improvement at final F-UP, while the MRI showed a significant tendency of repair tissue deterioration. Six failures	51
Di Martino et al Injury 2015 [20]	Prospective Case Series	23	38.0 ± 8.2 19 M/4F 25 ± 2.9	24	Chondral and osteochondral lesions in early OA	3.2 ± 1.9 cm^2^ 12 MFC/9 LFC/1 Tibia/ 1 Patella/6 Trochlea ICRS III—IV	MaioRegen (Finceramica)	Significant clinical improvement with better clinical results in younger patients. MOCART showed a complete integration of graft in 66.7% of cases. Two failures	38
Verdonk et al BJJ 2015 [73]	Prospective Case Series	38	30.5 ± 11.9 23 M/15F -	24	Osteochondral lesions	3.7 ± 2.4 cm^2^ 23 MFC/7 LFC/ 5 Patella/3 Trochlea ICRS III – IV	TruFit® (Smith&Nephew)	Significant improvement of clinical scores and MOCART score at final F-UP. Two failures	48
Berruto et al AJSM 2014 [6]	Prospective Case Series	49	37 ± 14 37 M/12F -	24	Osteochondral lesions	4.4 ± 1.3 cm^2^ 33 MFC/ 11 LFC/ 1 Trochlea/4 Tibia ICRS III-IV	MaioRegen (Finceramica)	Statistically clinical improvement at 2 years F-UP. MRI evaluation showed a complete filling of the lesion in 70% of cases. Three failures	55
Delcogliano et al Joints 2014 [16]	Prospective Case Series	23	24 19 M/4F 25.5 ± 7.7	24	OCD lesions	3.5 ± 1.4 cm^2^ 14 MFC/9 LFC ICRS III—IV	MaioRegen (Finceramica)	Promising stable results at short-term F-UP MRI scans showed complete filling of the defect in 80% of cases. No failures	54
Delcogliano et al KSSTA 2014 [15]	Prospective Case Series	19	26 ± 8 16 M/5F -	24	Osteochondral lesions	5.2 ± 1.6 cm^2^ 10 MFC/7 LFC/3 Tibia ICRS III—IV	MaioRegen (Finceramica)	Promising stable results at short-term F-UP Two failures	44
Gelber et al The Knee 2014 [29]	Prospective Case Series	57	36 (25–53) 51 M/6F 25 ± 3	45	Chondral and osteochondral lesions	3.6 cm^2^ (2.8—3.9) 22 MFC/15 LFC/ 20 Trochlea ICRS III—IV	TruFit® (Smith&Nephew)	Clinical scores improved at final F-UP, but alterations of subchondral bone and lamina were observed at MRI evaluation. One failure	54
Hindle et al KSSTA 2014 [36]	Retrospective Comparative Study	35 (66)	38.6 ± 13.3 M/F 27.9 ± 5.4	22	Chondral and osteochondral lesions	- - -	TruFit® (Smith&Nephew) vs Mosaicplasty	Mosaicplasty group had a higher rate of returning to sport and higher KOOS score compared to scaffold group. Five failures in the scaffold group	24
Kon et al AJSM 2014 [41]	Prospective Case Series	27	34.9 ± 10.2 18 M/9F -	60	Chondral and osteochondral lesions	2.9 ± 1.3 cm^2^ 7 MFC/5 LFC/11 Patella/ 7 Trochlea/2 Tibia ICRS III—IV	MaioRegen (Finceramica)	A statistically significant improvement of all scores at final F-UP, although not correlated with MRI results. Failures not reported	47
Kon et al J Mater Sci Mater Med 2014 [43]	Prospective Case Series	79	31.0 ± 11.3 63 M/16F -	24	Chondral and osteochondral lesions	3.2 ± 2 cm^2^ 41 MFC/26 LFC/ 15 Trochlea III-IV	MaioRegen (Finceramica)	A statistically significant improvements in all scores although not correlated with MRI findings. Three failures	49
Kon et al Injury 2014 [46]	Prospective Case Series	11	37.3 ± 11.0 6 M/5F 21.6 ± 2.6	24	Osteochondral lesions	5.1 ± 2.7 cm^2^ 2 Femur/11 Tibia III – IV	MaioRegen (Finceramica)	Clinical scores significantly improved at final F-UP of 2 years. No failures	34
Filardo et al AJSM 2013 [24]	Prospective Case Series	27	25.5 ± 7.7 19 M/8F 23.0 ± 2.7	24	OCD lesions	3.4 ± 2.2 cm^2^ 17 MFC/10 LFC ICRS III—IV	MaioRegen (Finceramica)	Good clinical outcome at 2-year of F-UP. Less favorable findings obtained with MRI. Failures not reported	43
Joshi et al AJSM 2012 [37]	Prospective Case Series	10	33.3 (16–49) 4 M/6F 28.8 (23–37)	24	Osteochondral lesions	2.6 cm^2^ (1–5) Patella III-IV	TruFit® (Smith&Nephew)	Good clinical results at 12 months in knee without concomitant injuries, with a worsening after 18 months of F-UP. Seven failures	55
Kon et al AJSM 2011 [38]	Prospective Case Series	28	35.3 ± 10.2 19 M/9F -	24	Chondral and osteochondral lesions	2.9 ± 1.3 cm2 8 MFC/5 LFC/12 Patella/ 7 Trochlea/2 Tibia ICRS III – IV	MaioRegen (Finceramica)	Clinical scores significantly improved at final F-UP of 2 years. Two failures	48

*BMI* Body mass index, *CMS* Modified Coleman Methodology Score, *F* Female, *F-UP* Follow-up, *ICRS* International Cartilage Repair Society score, *KOOS* Knee Injury and Osteoarthritis Outcome score, *LFC* Lateral femoral condyle, *M* Male, *MFC* Medial femoral condyle, *MOCART* Magnetic Resonance Observation of Cartilage Repair Tissue score, *MRI* Magnetic resonance imaging, *OA* Osteoarthritis, *OCD* Osteochondritis dissecans, *SPONK* Spontaneous osteonecrosis of the knee

### Qualitative data synthesis

Among the 34 articles included in the qualitative data synthesis, the evaluation of the study type showed only one randomized clinical trial (RCT), three retrospective/prospective comparative studies, and 30 case series (Table 1). Regarding the type of osteochondral scaffold investigated, 20 studies analyzed the results of MaioRegen (Finceramica, Faenza, Italy) with 19 case series and one RCT versus microfracture technique, 11 studies described the results of TruFit® (Smith & Nephew, Andover, MA) with nine case series and two retrospective comparative study (versus OAT and microfracture technique, respectively), and three studies reported the results of Agili-C™ (CartiHeal (2009) Ltd, Israel) with two case series and one retrospective study comparing two different versions of the same scaffold.

**Fig. 3:**
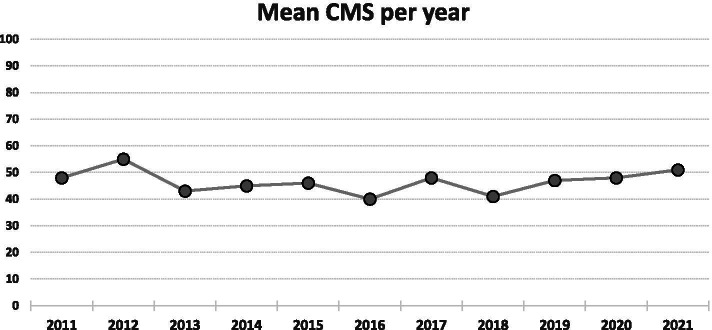
Trend over time of the mean modified Coleman Methodology Score (CMS) per year of the included articles on osteochondral scaffolds for the treatment of knee lesions

The evaluation with the CMS showed an overall poor quality of the included studies, with a mean of 44.4 ± 9.8 (range 24—67). Only two studies scored higher than 60, seven studies reached a score between 50 and 59, whereas 16 studies had a score between 40 and 49, and nine studies obtained a score lower than 40. No improvement over time was found for the CMS score of the published articles, as reported in Fig. 3. There was a 68% agreement between the two authors involved in the evaluation of CMS.

A total of 1022 patients were treated with cell-free osteochondral scaffolds (522 with MaioRegen, 304 with TruFit®, and 196 with Agili- C™), with a mean age of 35.6 ± 9.9 years (range 16.2—64.4). Patients were evaluated at a mean follow-up of 35.2 ± 19.3 months. In particular, 19 studies reported the outcome at short-term follow-up (≤ 24 months), eight at short/mid-term follow-up (24–60 months), and seven at mid/long-term follow-up (≥ 60 months), including the longest mean follow-up available in the literature (121 months) [69]. A wide range of heterogeneous clinical scores were used to evaluate patients. The most commonly used scores were: IKDC subjective score (25 articles), Tegner score (22 articles), IKDC objective score (16 articles), Knee Injury and Osteoarthritis Outcome Score (KOOS, 14 articles), Lysholm score (10 articles), and Visual Analogue Score (VAS) for pain (seven articles). Twenty-seven studies also reported an imaging evaluation, with the Magnetic Resonance Observation of Cartilage Repair Tissue (MOCART) score used in 24 studies (MOCART 2.0 in two studies) to evaluate scaffold integration and defect filling [66]. Due to the limited outcome data available, a quantitative data analysis was performed on the two most common scores reported at the follow-up times (IKDC subjective score and Tegner score).

### Safety and failures

Adverse events were documented in 23 of the included studies for a total of 806 evaluated patients. Most of these were mild symptoms (knee pain and effusion) complained by the patients and some cases of post-operative fever solved in a few days. Moreover, 13 cases of knee effusion that required knee arthrocentesis were described [68]. Different severe adverse events, requiring hospitalization or intervention to prevent permanent impairment or damage, were reported. In particular, post-operative joint stiffness was documented in 33 patients (4.3%) and required a knee mobilization under narcosis or an arthroscopic release to improve knee functionality (2.1% for TruFit®, 2.0% for Agili-C™, and 5.8% for MaioRegen, respectively). In studies evaluating TruFit® implantation, Gelber et al. [29] described one case of deep vein thrombosis and one case of acute septic arthritis solved after arthroscopic implant removal combined to specific antibiotic therapy over a period of six week. Hindle et al. [36] reported one case of suspected infection (developing a liquefied hematoma at one month post-operatively, and then solved after a six week of antibiotic therapy). Finally, Wang et al. [74] treated one patient presenting deep infection with irrigation and debridement.

A total of 65 failures was reported in the included studies, for an overall 7.0% failure rate at a mean follow-up of 31.0 months. Patients who failed underwent implant removal and subsequent alternative cartilage treatment or a partial/total knee replacement (36 and 29, respectively). Considering the failures reported in each scaffold group, patients treated with TruFit® had an overall 9.9% failure rate at a mean follow-up of 39.8 months, patients treated with Agili-C™ had an overall 8.2% failure rate at a mean follow-up of 18.0 months, and patients treated with MaioRegen had an overall 4.6% failure rate at a mean follow-up of 28.4 months.

### Quantitative data analysis

Among the 28 studies evaluating IKDC subjective or Tegner scores, six studies were excluded from the meta-analysis for the following reasons: no standard deviation reported (four studies) or no pre-operative clinical data reported (two studies). Thus, a total of 22 studies evaluating MaioRegen or Agili-C™ was included in the quantitative synthesis.

**Fig. 4:**
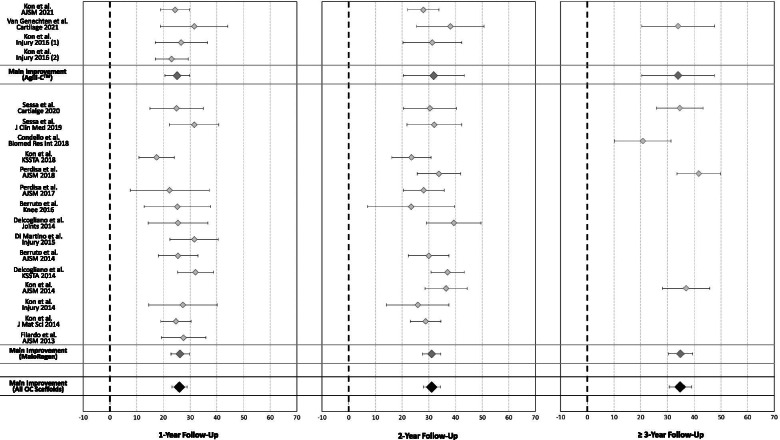
Forest plot of the mean improvement of the International Knee Documentation Committee (IKDC) subjective score at 1, 2, and ≥ 3 years’ follow-up

The IKDC subjective score was available for 640 patients in 18 studies (14 for MaioRegen and 4 for Agili-C™). In detail, 1-year follow-up was available for 565 patients (16 studies), 2-year follow-up for 476 patients (15 studies), and ≥ 3-year follow-up (mean 55.2 ± 13.7) for 113 patients (5 studies). Compared with the basal score, the meta-analysis showed a mean improvement of 26.0 (95% CI 23.3–28.8, I^2^ =  − 89%) at 1-year follow-up, 31.1 (95% CI 28.0–34.3, I^2^ =  − 69%) at 2-year follow-up, and 34.8 (95% CI 30.6–39.1, I^2^ =  − 59%) at ≥ 3-year follow-up (Fig. 4), all significantly higher than the baseline (*p* < 0.05), with a further statistically significant improvement from 1 to 3 years of follow-up (*p* = 0.003).

**Fig. 5:**
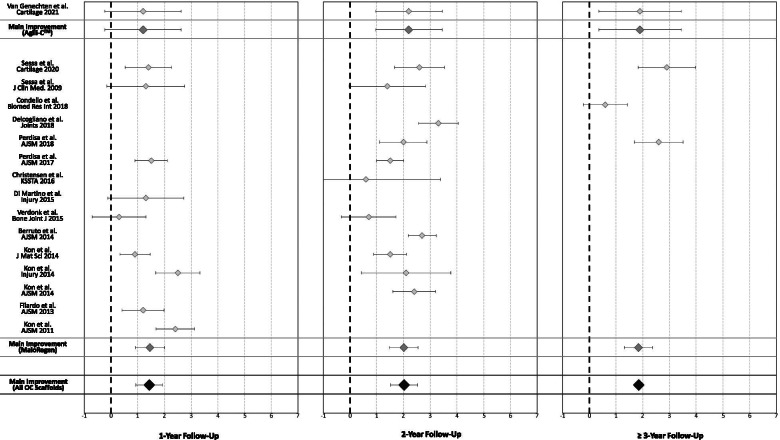
Forest plot of the mean improvement of the Tegner score at 1, 2, and ≥ 3 years’ follow-up

The activity level evaluated with Tegner score was available for 499 patients in 16 studies (15 for MaioRegen and one for Agili-C™). In detail, 1-year follow-up was available for 341 patients (10 studies), 2-year follow-up for 347 patients (12 studies), and ≥ 3-year follow-up (mean 54.0 ± 15.5) for 86 patients (4 studies). Compared with the basal score, the meta-analysis showed a mean improvement of 1.4 (95% CI 0.9–1.9, I^2^ =  − 40%) at 1-year follow-up, 2.0 (95% CI 1.5–2.5, I^2^ =  − 50%) at 2-year follow-up, and 1.9 (95% CI 1.4–2.4, I^2^ =  − 80%) at ≥ 3-year follow-up (Fig. 5), all significantly higher than baseline (*p* < 0.05), but without any significant difference among follow-up times.

In Figs. 4 and 5, the results have been reported separately for MaioRegen and Agili-C™ scaffolds.

## Discussion

The main finding of this study is that the available literature supports the use of multi-layer cell-free scaffolds for the treatment of patients with knee osteochondral defects. These scaffolds provided promising clinical improvement at short/mid-term follow-up, with a low rate of adverse events and an overall failure rate of 7.0% at a mean 31.0 months of follow-up. Nevertheless, the evidence level of the available studies was limited and high-level trials at longer follow-up are still missing.

Among the developed osteochondral scaffolds, only three have currently been documented in clinical trials. TruFit® was the first one introduced in the clinical practice. It is a bilayer scaffold made of a semiporous poli-lactic (PLGA), poli-glycolic acid (PGA), and calcium sulfate biopolymer. The different size cylinders (from 5 to 11 mm and with a maximum depth of 18 mm) were initially introduced with the indication to backfill graft donor sites during OAT procedures, but they were later mainly used as a one-step osteochondral treatment [5]. MaioRegen is the most widely studied multi-layer scaffold. It is a nanostructured implant consisting of different ratios of collagen and hydroxyapatite organized in three-layers. The composition of this scaffold reproduces the extracellular matrix structures of cartilage and bone tissues and is based on the nucleation of hydroxyapatite nanocrystals onto self-assembled collagen fibers to generate a chemically and morphologically graded biomimetic material [71]. Initially, this scaffold was obtained from a square of 35 × 35 mm (manual sizing) with a depth of 6 ± 2 mm. Currently, the scaffold is available also in a cylindrical shape with different sizes (12—18 mm) and different depths (2 to 6 mm). Agili-C™ is the most recent osteochondral scaffold studied in the clinical practice. It is an aragonite-based scaffold consisting of two layers: a bone phase made of calcium carbonate in the aragonite crystalline form, and a superficial cartilage phase composed of modified aragonite and hyaluronic acid. This scaffold was developed in the shape of cylinders, with different sizes in terms of width and depth. Recently, a tapered version of the implants, with an angle of 2 degrees from the longitudinal axis, has been designed to improve the press-fit implantation of the cylinder [47].

These multi-layer scaffolds have been investigated in several clinical studies for the treatment of osteochondral defects of the knee. Studies investigating the safety and effectiveness of the TruFit® implant often reported poor outcomes in terms of clinical results, failure rate, and histological evaluation. Dhollander et al. [19] documented a failure rate of 20% at 1 year of follow-up with the histological analysis showing fibrous vascularized repair tissue. Shivji et al. [69] evaluated this scaffold also at long-term follow-up (121 months), reporting no statistically significant improvement in any score from baseline, while the MRI evaluation showed incomplete or no evidence of plug incorporation and persistent chondral loss. Based on these poor results, the TruFit® scaffold has been withdrawn from the market. Regarding the scaffolds still available in the clinical practice, since the first trial published in 2011, numerous studies showed promising results in terms of safety profile and clinical improvement [38]. In particular, this meta-analysis demonstrated that at 1-year follow-up the IKDC subjective score improved significantly compared with the baseline scores, demonstrating the efficacy of these techniques. Moreover, the evaluation at follow-up showed a further improvement from 1 to 3 years, suggesting that most of the benefit is achieved in the first year, but also that the osteochondral regeneration might need more time to reach stable results. This seems to be different from the trend previously reported for cell-free chondral scaffolds, as in a recent meta-analyses stable results were documented after 1 year using chondral cell-free matrices for the treatment of knee cartilage lesions [3, 70]. Still, despite the more complex lesion pattern due to the subchondral bone involvement, the osteochondral scaffolds provided a satisfactory clinical improvement. Unfortunately, the current literature does not allow to draw conclusions on the long-term results for osteochondral scaffolds, with no studies investigating the results over six years.

Another important aspect evaluated in this study was the activity level. Sport represents a fundamental parameter to consider for cartilage lesions, especially in young and active patients [2]. The meta-analysis on the Tegner activity level documented a significant improvement from baseline to 1-year follow-up, whit stable values at the at 2-year and at ≥ 3-year follow-up evaluations. This stable trend is in line with that reported for the other cartilage procedures, ranging from autologous chondrocyte implantation (ACI) to OAT [10, 65]. Conversely, some authors described a significantly deterioration in activity level from 2 years of follow-up after microfracture technique [10, 30, 33]. However, this meta-analysis reported a lower mean improvement of the Tegner score at 1- and 2-year follow-ups compared with the literature values offered by microfracture, OAT and ACI procedures at the same follow-ups [49]. The lower results in term of activity level found for the osteochondral scaffold could be due to the complexity of the treated lesions affecting the entire osteochondral unit and the heterogeneity of the investigated populations, which involved several complex cases ranging from early OA patients to knee osteonecrosis [7, 12, 39, 67]. For example, Maioregen scaffold was also used to address complex lesions, and the most recent article of Agili-C™ was targeted to the complex population of patients with OA joints, challenging conditions where other procedures focused only on the cartilage layer failed to provide satisfactory outcomes [26, 28, 39]. Further studies should explore if the lower activity level reached in these studies is due to the more challenging treatment indications of osteochondral scaffolds, or to a lower regenerative potential of these osteochondral scaffolds versus the chondral and cell-based treatments largely documented in the literature of the last two decades.

Besides the clinical results, most of the included studies also performed an imaging assessment, in order to evaluate the scaffold maturation over time. MRI evaluation demonstrated controversial and heterogeneous findings. On one side, some studies described complete filling of the cartilage layer and a good integration of the graft, on the other side, most of the included studies reported the presence of subchondral bone alterations after the scaffold implantation [11, 40]. Moreover, the maturation of the scaffold appeared slow, especially in the subchondral bone area, even though the majority of the studies highlighted a positive evolution over time. In fact, several authors reported a significant increase of the defect filling and a significant subchondral bone status improvement over time, although with persistent signal abnormalities over time [40, 68]. However, no correlation was found between imaging findings and clinical outcomes. The persistence of an altered signal and a slow maturation process of the subchondral layer suggest that further improvements are still needed to obtain better tissue regeneration and optimal durable clinical results [44].

The systematic review and meta-analysis also underline the overall low-quality level of the studies in this field, with only one RCT and three retrospective/prospective comparative studies. In a retrospective comparative study, Wang et al. [74] compared the results of TruFit® scaffold to microfracture for the treatment of 132 patients with knee chondral or osteochondral defects. While no significant differences in clinical outcomes were reported up to 5 years, the scaffold group reported better activity level and MRI appearance of the defect, resulting in a more frequent good-quality tissue fill and cartilage isointensity. The same scaffold has been also compared with osteochondral mosaicplasty in a retrospective study conducted by Hindle et al. on 66 patients with knee articular cartilage defects [36]. At the final follow-up (22 months for the scaffold group and 30 for the mosaicplasty group), the authors demonstrated significantly better clinical outcomes and a higher rate of return to sport after mosaicplasty, indicating that mosaicplasty may be more effective than TruFit® approach. In the only RCT available in this field, Kon et al. [40] evaluated 100 patients affected by symptomatic knee chondral and osteochondral lesions treated with MaioRegen or microfracture. While comparable results were found in the overall population, the osteochondral scaffold provided significantly better clinical results compared to microfracture in the treatment of deep osteochondral lesions and sport active patients at 2 years of follow-up. The authors concluded that microfracture technique can be considered a treatment option for purely chondral lesions, while offers worse results for osteochondral lesions, where osteochondral scaffolds showed to be a more suitable therapeutic solution.

This study presents some limitations, that reflect the weakness of the literature on the field. In fact, the lack of studies at long-term follow-up needs to be underlined, particularly given that the earliest scaffolds should have already reached a long-term follow-up with relatively numerous cohorts of patients. The overall quality level of the included studies is generally low, as confirmed by the low CMS and by the presence of only 1 RCT and 3 comparative studies. Moreover, the analysis of the CMS did not show any improvement over time. Moreover, only a small percentage of studies evaluated the treatment of isolated osteochondral lesions, while the treatment was often performed in association with other procedures, given the complexity of patients treated with osteochondral scaffold. Finally, numerous and heterogeneous scores were adopted, therefore hindering the possibility of comparison among studies. Accordingly, a weakness of the meta-analysis is represented by the low number of patients included and the high proportion of level IV studies. Despite the heterogeneous patient populations, lesion locations, the different scaffold used, and the different follow-up times, an overall short- to mid-term benefit was described for the two osteochondral scaffolds currently available in the clinical practice. Further high-level studies with longer follow-up are needed, as well as comparative trials with the other osteochondral procedures including mosaicplasty and OCA, to clarify the potential and indication of these techniques to restore a functional osteochondral unit. Moreover, comparative studies among osteochondral scaffolds could help improving the field. Finally, while these cell-free scaffolds have been developed to overcome the problems related to cell-expansion, the possibility to augment them with cell concentrates, to exploit their regenerative and homeostatic potential [27, 55, 63], in one-step procedures should be explored to help addressing challenging lesions such as those presenting an OA joint environment.

## Conclusions

The current literature suggested that multi-layer osteochondral scaffolds may provide clinical benefits for the treatment of knee osteochondral lesions at short- and mid-term follow-up and with a low number of failures, although the sport activity level obtained seems to be limited. Further research with high-level studies is needed to confirm the role of multi-layer scaffold for the treatment of osteochondral lesions of the knee.

## Data Availability

Not applicable.

## Abbreviations

ACI: Autologous chondrocyte implantation
BMI: Body mass index
CI: Confidence Interval
CMS: Modified Coleman Methodology Score
ICRS: International Cartilage Repair Society score
KOOS: Knee Injury and Osteoarthritis Outcome score
MOCART: Magnetic Resonance Observation of Cartilage Repair Tissue score
MRI: Magnetic resonance imaging
OA: Osteoarthritis
OAT: Autologous osteochondral transplantation
OCA: Osteochondral allograft transplantation
PGA: Poli-glycolic acid
PLGA: Poly lactic-*co*-glycolic acid
PRISMA: Preferred Reporting Items for Systematic Reviews and Meta-Analysis
RCT: Randomized clinical trial
VAS: Visual Analogue Score
